# Comparison of Renal Function and Other Health Outcomes in Vegetarians versus Omnivores in Taiwan

**DOI:** 10.3329/jhpn.v28i5.6155

**Published:** 2010-10

**Authors:** Chih-Kuang Lin, Deng-Juin Lin, Chi-Hwa Yen, Shiuan-Chih Chen, Chun-Chieh Chen, Tsun-Yen Wang, Ming-Chih Chou, Horng-Rong Chang, Meng-Chih Lee

**Affiliations:** ^1^ Institute of Medicine, Chung Shan Medical University, Taichung 402, Taiwan; ^2^ Division of Nephrology, Department of Internal Medicine, Chung Shan Medical University Hospital, Taichung 402, Taiwan; ^3^ School of Medicine, Chung Shan Medical University, Taichung 402, Taiwan; ^4^ Bureau of Health, Taichung City, Taiwan; ^5^ Center for Education and Research on Geriatrics and Gerontology, Chung Shan Medical University, Taichung 402, Taiwan; ^6^ Department of Family and Community Medicine, Chung Shan Medical University Hospital, Taichung 402, Taiwan

**Keywords:** Cross-sectional studies, Diet, Diet, Vegetarian, Renal function, Taiwan

## Abstract

Renal disease is one of the top 10 leading causes of death, and the incidence of end-stage renal disease in Taiwan is the highest in the world. Many dietitians consider the diet of plant origin consumed by vegans to be ‘lighter’ and ‘more healthful’ than the diet of both plant and animal origin consumed by omnivores. Dietary protein has significant effects on renal functions. The study explored the effects of both the diets on renal functions. The study subjects included 102 Buddhist nun vegetarians and an equal number of matched control group (omnivores). A cross-sectional study was performed to investigate the effects of the diet of plant origin and the diet of both plant and animal origin on renal functions. There was no difference in the renal functions between the two groups. However, systolic blood pressure, blood urea nitrogen, serum sodium, glucose, cholesterol levels, and urinary specific gravity were lower in the vegetarian group. Although these results were compatible with general concepts regarding diet of plant origin, after adjusting for age, the duration of intake of this diet had no effect on the renal functions. Based on the findings, it is concluded that the renal functions, in terms of the estimated glomerular filtration rate, were not different between the vegetarians and the omnivores.

## INTRODUCTION

In the last few years, renal disease has become one of the 10 leading causes of death in Taiwan. Results of an analysis of the United States Renal Data System (USRDS) showed that the incidence rates of reported end-stage renal disease (ESRD) in 2007 were the highest in Taiwan, at 415 per million population, followed by Mexico, the United States, Japan, and Turkey, at 372, 361, 285, and 229 per million population respectively (1). In 2009, the Department of Health, Taiwan, reported that mortality due to nephritis, nephrotic syndrome, and other kidney diseases was the 10th leading cause of death in Taiwan (2).

The diet of plant origin has become popular in Taiwan because many perceive it as a ‘lighter’ alternative and is, therefore, more healthful. Many studies focused on the impact of a diet of plant origin on nutrition and health (3). Hyperlipidaemia, persistent proteinuria, glomerular hyperfiltration, and hypertension may contribute to the deterioration of renal functions. In addition, excess protein may also exacerbate renal functions, especially with diabetic nephropathy. Results of some studies, using animal and human models, suggest that restrictions of dietary protein can significantly retard the progression of chronic renal insufficiency (4–6). Diet of vegetarians rich in plant proteins, especially soy-proteins, has positive effects on blood pressure and lipid profiles. These positive effects include an improvement in proteinuria, hyperfiltration and renal perfusion, and decreased renal injury. To prevent or delay further progression of kidney damage in diabetic nephropathy, results of studies suggest replacing animal protein with soy-protein (7–9). However, the impact of diet of the vegetarians on protecting renal function remains unclear. The aim of this study was to explore the effects of diets of the vegetarian and omnivorous consumers on renal functions.

## MATERIALS AND METHODS

### Study subjects

During March 2006–June 2007, we screened chronic diseases in Taichung City in central Taiwan. Initially, we recruited 105 Buddhist nuns, aged 20-78 years, from a temple. Of the 105 nuns, 102 were enrolled with complete data. The average history of practising diet of plant origin by the subjects was 17.8 years. Omnivorous female subjects were selected through a simple random-sampling method. The study used a 1:1 ratio of age, body mass index (BMI), and education level-matched vegetarian to omnivorous female subjects. The final number of the study subjects included 102 vegetarians and 102 omnivores. Subjects diagnosed with chronic renal diseases in both vegetarian and non-vegetarian groups were excluded.

### Biochemical analysis

We collected fasting blood samples for studying both routine biochemical screening of renal disorders and lipid profile. The study used the Olympus AU-2700 (Olympus Japan Co. Ltd., Tokyo, Japan) and the SYSMEX XE-2100 (TOA Medical Electronics, Kobe, Japan) to measure the biochemical components. These measurements included blood urea nitrogen (BUN), serum creatinine, sodium, potassium, chloride, calcium, phosphorus, uric acid, albumin, fasting plasma glucose, triglycerides, and total cholesterol. The Medical Laboratory of the Chung Shan Medical University Hospital analyzed all the samples.

### Definition of terms

We used serum creatinine, age, and gender to calculate the estimated glomerular filtration rate (eGFR) according to the equation of simplified modification of diet in renal disease (MDRD) (10). Table 1 classifies the renal functions into five stages. This aligns with the national DOQI (Disease Outcomes Quality Initiative) standards.

**Table 1.** Classification of chronic kidney disease (11)

Stage	Description	eGFR (mL/minute/ 1.73 m^2^)
1	Kidney damage with normal or elevated GFR	>90
2	Kidney damage with mild decreased GFR	60-89
3	Moderate decreased GFR	30-59
4	Severe decreased GFR	15-29
5	Renal failure	<5 (or dialysis)

eGFR=Estimated glomerular filtration rate

The Bureau of Health standards for Taiwan considers a BMI of >24 to be normal and BMI of <18 to be abnormal. Systolic blood pressure of >130 mmHg and/or diastolic blood pressure of >80 mmHg is defined as high blood pressure. Hyperglycaemia is defined as fasting plasma glucose of >100 mg/dL while hypertriglyceridaemia is defined as serum triglycerides of >150 mg/dL. Hypercholesteraemia is the serum total cholesterol of >200 mg/dL. Serum creatinine and BUN are elevated if the serum levels are >1.3 mg/dL and >20 mg/dL respectively. A serum sodium level of >145 mEq/L is seen as hypernatraemia. Hyperuricaemia is serum uric acid of >6 mg/dL. Abnormal urine is an elevated specific gravity (>1.020) of urine, proteinuria, haematuria, and pyuria when urine dipstick chemical analysis showed 1+ of protein, occult blood, or leukocyte esterase.

### Statistical analysis

The study used descriptive analysis, *t*-test, regression model, and chi-square analysis. Data were analyzed using the SPSS software (version 12).

## RESULTS

### Demographics of vegetarians and omnivores

Each group had 102 female subjects, and their mean age was 46.6 years (Table 2). Of the subjects, 27.5%, 39.2%, 27.5%, and 13.7% were aged <39, 40-49, 50-59, and >60 years respectively. Underweight subjects accounted for 2.5% while 60.8% were of normal weight, and 36.8% were overweight. 75.0% of the subjects had mildly decreased eGFR, 4.9% moderate decreased eGFR, 0.5% severe decreased eGFR, 0.5% end-stage renal failure, and 18.6% of the subjects had normal eGFR.

**Table 2.** Basic characteristics and performance of kidney function

Characteristics	Vegetarians (n=102)		Omnivores (n=102)		Total	
	No.	%	No.	%	No.	%
Age (years)*						
<39	28	27.5	28	27.5	56	27.5
40-49	40	39.2	40	39.2	80	39.2
50-59	20	20.5	20	20.5	40	20.5
>60	14	13.7	14	13.7	28	8.8
Educational level*						
College and above	30	33.3	34	29.4	64	31.4
Junior and senior high school	41	40.2	45	44.1	86	42.1
Primary school	27	26.5	27	26.5	54	26.5
BMI*						
<18	3	2.9	2	2.0	5	2.5
18-24	63	61.8	61	59.8	124	60.8
>24	36	35.3	39	38.2	75	36.8
eGFR (mL/minute/1.73 m^2^)*						
<15	1	1.0	0	0.0	1	0.5
15-29	1	1.0	0	0.0	1	0.5
30-59	4	3.9	6	5.9	10	4.9
60-89	77	75.5	76	74.5	153	75.0
>90	19	18.6	20	18.6	39	18.6

*p>0.10; BMI=Body mass index; eGFR=Estimated glomerular filtration rate

### Characteristics of omnivores and vegetarians

Table 3 shows that there was no significant difference in the BMI between the groups. The systolic blood pressure was significantly lower in the vegetarian group compared to the omnivorous group (114.8±15.7 vs 119.7±18.5, p=0.04), although the diastolic blood pressure of vegetarians was higher compared to the omnivores (77.0±10.0 vs 73.3±10.1, p=0.01), indicating a narrow range between systolic blood pressure and diastolic blood pressure in the vegans. The BUN, BUN/creatinine ratio, total cholesterol, and fasting plasma glucose levels were all significantly lower (p<0.05) in the vegetarian group. However, creatinine, uric acid, and triglyceride levels were not different. The eGFR was 79.6 mL/minute/1.73 m^2^ in the vegetarian group and 79.0 mL/minute/1.73 m^2^ in the omnivorous group without any significant difference.

**Table 3.** Comparison of characteristics between vegetarians and omnivores

Variable	Vegetarians (n=102) mean±SD	Omnivores (n=102) mean±SD	p value
Age (years)	46.6±11.3	45.5±13.4	0.50
Body mass index	23.4±3.9	23.3±3.5	0.73
Systolic blood pressure (mmHg)	114.8±15.7	119.7±18.5	0.04*
Diastolic blood pressure (mmHg)	77.0±10.0	73.3±10.1	0.01*
BUN (mg/dL)	10.9±4.8	13.8±4.6	<0.001*
Creatinine (mg/dL)	0.9±0.3	0.9±0.1	0.75
BUN/creatinine ratio	12.7±3.7	16.1±4.5	<0.001*
Sodium (mEq/L)	139.0±7.4	144.3±2.3	<0.001*
Uric acid (mg/dL)	4.8±1.0	4.8±1.2	0.92
Fasting plasma glucose (mg/dL)	76.6±28.2	88.6±21.4	0.001*
Trigycerides (mg/dL)	123.7±78.5	104.2±70.4	0.06
Cholesterol (mg/dL)	168.8±33.0	186.6±31.8	<0.001*
eGFR (mL/minute/1.73 m^2^)	79.6±14.7	79.0±14.4	0.78

*p<0.05; BUN=Blood urea nitrogen; eGFR=Estimated glomerular filtration rate; SD=Standard deviation

### Abnormal levels in vegetarian and omnivorous groups

Table 4 shows that there were no significant differences in the elevated BMI or elevated blood pressure.

**Table 4.** Comparison of abnormal parameters between vegetarians and omnivores

Unusual situation	Vegetarians (n=102)		Omnivores (n=102)		p value
	No.	%	No.	%	
Elevated BMI	39	38.2	41	40.2	0.74
Elevated blood pressure					
SBP >140 mmHg	9	8.8	15	14.7	0.19
DBP >90 mmHg	8	7.8	2	2.0	0.05
Elevated BUN	3	2.9	6	5.9	0.34
Elevated creatinine	2	2.0	1	1.0	0.56
Hypernatraemia	0	0.0	30	29.4	<0.001*
Hyperuricaemia	10	9.8	12	11.8	0.65
Hyperglycaemia	8	7.8	12	11.8	0.35
Hypertriglycaeridaemia	25	24.5	15	14.7	0.08
Hypercholesteraemia	16	15.7	30	29.4	0.02*
eGFR <90	83	81.4	82	80.4	0.86
Elevated urinary specific gravity	41	40.2	78	76.5	<0.001*
Protaeinuria	10	9.8	4	3.9	0.10
Haematuria	31	30.4	27	26.5	0.54
Pyuria	13	12.7	11	10.8	0.66
Bactaeriuria	53	52.0	42	41.2	0.12

χ^2^ test:

*p<0.05; BMI=Body mass index; BUN=Blood urea nitrogen; DBP=Diastolic blood pressure; eGFR=Estimated glomerular filtration rate; SBP=Systolic blood pressure

The proportion of subjects with hypernatraemia and hypercholesteraemia was significantly (p<0.05) lower in the vegetarian group. Other biochemical parameters, including hyperuricaemia, hyperglycaemia, hypertriglyceridaemia, and eGFR, did not show any significant differences.

The proportion of subjects with elevated urinary specific gravity was significantly lower in the vegetarian group (40.2% vs 76.5%, p<0.05). However, other parameters, including proteinuria, haematuria, pyuria, and bacteriuria, were not different.

### Duration of diet intake by vegetarians and parameters of health

The results indicated that the diet of vegetarians might have affected some parameters. Table 5 presents the results on analysis of the effects of the duration of diet intake by vegetarians on health parameters. The age of a vegetarian significantly (p<0.05) correlated with BUN, cholesterol, and eGFR level. Blood pressure was significantly (p<0.05) associated with creatinine level. In addition, fasting plasma glucose positively correlated with uric acid and triglyceride levels (p<0.05). The duration of diet intake by vegetarians was not associated with any health markers in the study.

**Table 5.** Predictors and parameters of renal functions and health

Response variable	Independent variable											
	Age		Duration of diet intake by vegetarians		Systolic blood pressure		Diastolic blood pressure		Fasting plasma glucose		Constant	
	β	p value	β	p value	β	p value	β	p value	β	p value	β	p value
BMI	-0.01	0.78	0.02	0.63	0.07	0.07	0.03	0.60	0.02	0.19	12.27	<0.0001
BUN	0.20	<0.0001*	0.001	0.97	0.01	0.63	-0.03	0.37	-0.01	0.22	3.30	0.19
Creatinine	0.003	0.08	0.002	0.20	0.003	0.04*	-0.004	0.03*	-0.0001	0.85	0.69	<0.0001
Sodium	0.05	0.36	0.06	0.26	-0.02	0.70	0.04	0.49	-0.01	0.57	135.6	<0.0001
Uric acid	0.01	0.36	0.01	0.30	0.02	0.09	-0.01	0.33	0.01	0.04*	2.82	0.001
Trigyceride	-0.20	0.78	1.21	0.11	0.38	0.52	0.97	0.26	1.51	<0.0001*	-124.1	0.03
Cholesterol	1.42	<0.0001*	-0.34	0.34	0.16	0.57	0.40	0.32	0.14	0.21	49.9	0.06
eGFR	-1.18	<0.0001*	-0.07	0.67	0.01	0.92	0.20	0.27	0.06	0.20	119.5	<0.0001

This study analyzed the response variables into the regression model to five independent variables (age, period of diet intake by vegetarians, systolic blood pressure, diastolic blood pressure, and fasting plasma glucose);

*p<0.05; BMI=Body mass index; BUN=Blood urea nitrogen; eGFR=Estimated glomerular filtration rate

## DISCUSSION

A few studies have explored the effects of diets consumed by vegetarians and omnivores on the nutrition status. Results of a study by Ko *et al*. in Taiwan showed blood pressure, cholesterol level, and glucose level to be significantly lower in female vegetarians when compared those in female omnivores. However, this was not evident in male subjects (12). The present study also revealed that blood pressure (systolic), cholesterol level, and glucose level were lower in the vegetarian group. The finding was in agreement with that from previous studies (13,14). In addition, BUN and sodium levels were also lower in the vegetarian group. There was no significant difference in BMI and creatinine, which confirms the findings of Chen *et al*. (15).

Diastolic blood pressure was significantly higher in the vegetarian group that was discordant with the previously-mentioned Ko study that demonstrated significance only in the female group (12). Therefore, more studies are needed to clarify the association between blood pressure level and vegetarian diet.

Another study in Thailand revealed a significantly-lower level of BUN, BUN/creatinine ratio, and a lower urinary protein excretion rate in vegetarians (16). Our study has shown similar results. In addition, the eGFR level was not significantly different between the groups. This suggests that a diet of the vegetarians does not have a significant effect on renal functions.

### Conclusions

The results of the present study showed that renal functions were not different between the vegetarians and the omnivores. Systolic blood pressure, BUN and sodium, glucose, cholesterol levels, and urinary specific gravity were lower in the vegetarian group. Although these results are compatible with general expectations, after adjusting for age, diet had no effect on renal function. On the other hand, there was a slightly-higher diastolic blood pressure in the vegetarian group. The vegetarians appeared to have lower systolic blood pressure, BUN, BUN/creatinine ratio, sodium, and fasting plasma glucose. Further research should continue to monitor and study the meanings of these parameters.
